# How to perform fluid de-escalation in critical care

**DOI:** 10.1016/j.aicoj.2026.100075

**Published:** 2026-05-06

**Authors:** Rahel E. Bircher, Alessandro Ostini, Carmen A. Pfortmueller, Anna S. Messmer

**Affiliations:** Department of Intensive Care Medicine, Inselspital, Bern University Hospital, University of Bern, Switzerland

**Keywords:** Fluid de-escalation, Fluid therapy, Critical care, Fluid de-resuscitation, Fluid accumulation, Diuretics, Renal replacement therapy

## Abstract

Fluid management is a core aspect of critical care, guiding decisions around the type, timing, and amount of fluid therapy. The administration of intravenous fluids aims to restore and maintain tissue perfusion, replace overt losses, and serve as a carrier for drug delivery. While fluid administration is often emphasised during the resuscitation phase, growing evidence highlights the risks associated with sustained positive fluid balance, including tissue oedema, organ dysfunction, and increased mortality.

A clear understanding of the pathophysiological mechanisms underlying fluid homeostasis can support decision-making during fluid de-escalation. Recognising the role of the lymphatic system in interstitial fluid clearance, and its potential impairment during critical illness, may help guide appropriate timing and realistic expectations for fluid removal.

Fluid de-escalation includes limiting fluids to daily physiological needs, and when excess fluid persists, active fluid removal using pharmacological or mechanical interventions may be required. Evidence supporting adjunctive measures, such as albumin, hypertonic saline, or compression techniques, remains limited. There is increasing interest in understanding whether treatment effects differ across patient phenotypes, and studies in patients with lung injury suggest this may also apply to fluid management strategies. In one study, hyperinflammatory patients appeared to benefit from a conservative fluid approach, while hypoinflammatory patients had worse outcomes, despite no overall difference in mortality between the strategies. These findings highlight the potential value of phenotype-guided fluid strategies.

Although fluid de-escalation is clinically important, evidence on the optimal timing, volume, and duration of fluid removal remains limited. While individualized ICU care often incorporates real-time hemodynamic variables, current strategies rely heavily on clinical judgment in the absence of standardized criteria. It also remains unclear whether a degree of permissive hemodynamic instability might be acceptable for preventing or reversing fluid overload.

Ultimately, weaning from fluid support should be seen as a continuous, individualized process. Ward round discussions should explicitly name fluid overload as a working diagnosis and recognise persistent fluid accumulation or difficulty in removal as a barrier to recovery, comparable to difficult weaning from mechanical ventilation or ICU-acquired weakness.

## Introduction

Intravenous fluid therapy is a fundamental component of critical care. Its primary objective is being the restoration and maintenance of tissue perfusion, which is an essential determinant of cardiac output, systemic blood pressure, and renal function [1]. Additionally, intravenous fluid serves as carrier for drug delivery, as replacement for overt fluid losses, and as a source of nutritional support.

Intravenous fluids are often conceptualised as pharmacologic agents, with established indications, contraindications, and a clearly defined profile of adverse effects [2]. This framework has substantially advance the rational use of fluids in critical care and provides an important foundation for strategies of de-escalation. Beyond a purely pharmacological perspective, fluid administration in critically ill patients may also be understood from a physiological standpoint. In acute circulatory failure, fluids are administered with the aim of temporarily maintaining or restoring organ perfusion and function. In the setting of acute circulatory failure, fluids are administered to temporarily maintain or restore organ perfusion and function. From this standpoint, fluid therapy can be regarded as a form of temporary organ support, aimed at sustaining organ perfusion rather than solely correcting haemodynamic variables [1].

This modality of organ support is essentially limited in two critical ways. First, its effects are often transient and inefficient, requiring continuous reassessment and frequent re-administration to maintain its intended physiological benefit [3,4]. However, the impact of fluids on circulatory and tissue-level perfusion tends to be short-lived, particularly in patients with ongoing capillary leak, systemic inflammation, or distributive shock. Second, fluid therapy is associated with a well-documented spectrum of adverse effects, particularly when administered in large quantities [5,6]. These include tissue oedema, impaired gas exchange, abdominal compartment syndrome, and worsened outcomes in various critical care populations [6,7].

Thus, while fluids can be appropriately classified within a pharmacologic framework, a more nuanced understanding considers them as a form of temporary organ support while underlying pathology is addressed. This approach demands the same level of timely weaning and withdrawal as other organ support strategies in critical care.

## Terminology and definitions

Fluid overload or fluid accumulation is defined as cumulative positive fluid balance resulting in a weight gain of more than 5–10% of baseline body weight [8].

Both terms *fluid de-escalation* and *fluid de-resuscitation* are used in the literature, though their definitions are not always consistent. In this review, we refer to fluid de-escalation as the broader strategy of fluid stewardship, encompassing the reduction or cessation of fluid administration after the initial resuscitation phase. In contrast, fluid de-resuscitation describes the more specific process of actively removing accumulated excess fluid, often aligned with concepts such as late goal-directed fluid removal and late conservative fluid management [2,9,10].

## Fluid clearance at the tissue level: key mechanisms

**Fig. 1:**
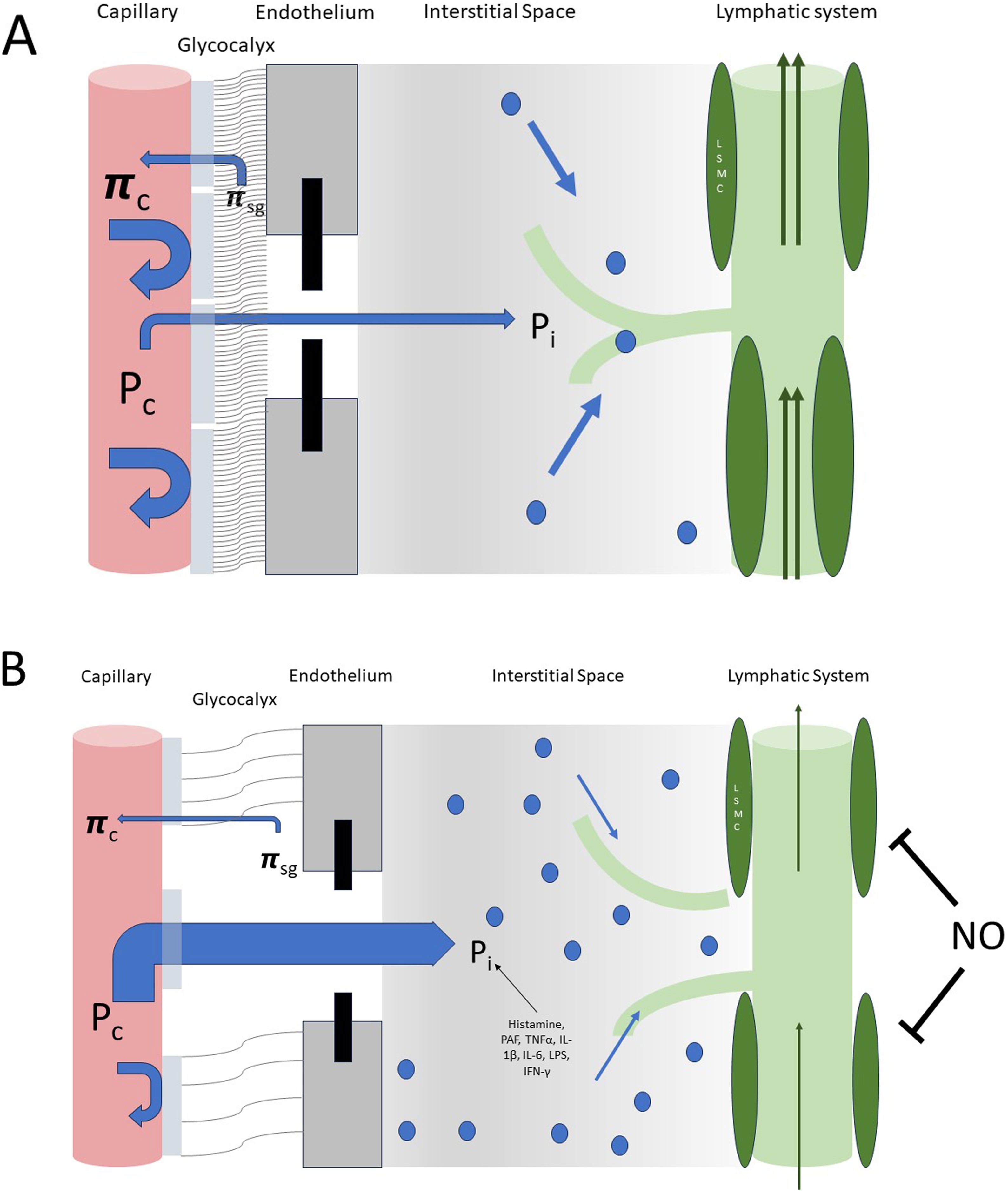
Fluid clearance at the tissue level. Panel A – Normal physiology. Fluid extravasation is driven by the hydrostatic pressure gradient between capillaries and the interstitial space. Most fluid resorption is facilitated by the lymphatic system, which actively transports interstitial fluid through coordinated peristaltic activity of LSMCs. Additionally, interstitial fluid is reabsorbed along an oncotic pressure gradient between the subglycocalyx space and the plasma. In response to increased fluid extravasation, lymphatic clearance is enhanced by upregulated peristalsis of the lymphatic vessels. Panel B – Critical Illness: Disruption of the glycocalyx and endothelial junctions increases vascular permeability and fluid extravasation. Cytokines reduce interstitial fluid pressure (Pi), while hypoalbuminemia and protein leakage into the subglycocalyx space diminish the oncotic gradient for resorption. Nitric oxide further impairs lymphatic contractions, reducing fluid clearance. Together, these factors promote interstitial fluid accumulation and oedema. **P**_c_, capillary hydrostatic pressure; **P**_i_, interstitial hydrostatic pressure; **π**_c_, capillary oncotic pressure; **π**_sg_, subglycocalyx oncotic pressure; **LSMC**, lymphatic scmooth muscle cells; **PAF**, platelet activation factor; **IL**, interleukin; **LPS**, lipopolysaccharides; **IFN-γ**, interferon-γ.

In recent years, the traditional Starling model of bidirectional fluid exchange across the microvasculature has been fundamentally revised [11]. New insights into microvascular biology, particularly the role of the endothelial glycocalyx, have changed how we understand capillary permeability and vascular barrier function [12,13]. The glycocalyx acts as both a physical and molecular barrier, so that fluid reabsorption happens across the sub-glycocalyx space rather than directly between plasma and interstitial fluid [6,14]. As a result, under normal conditions, little fluid is reabsorbed at the venous end of capillaries, and most filtered fluid is removed via the lymphatic system, see Fig. 1 (Panel A).

**Fig. 2:**
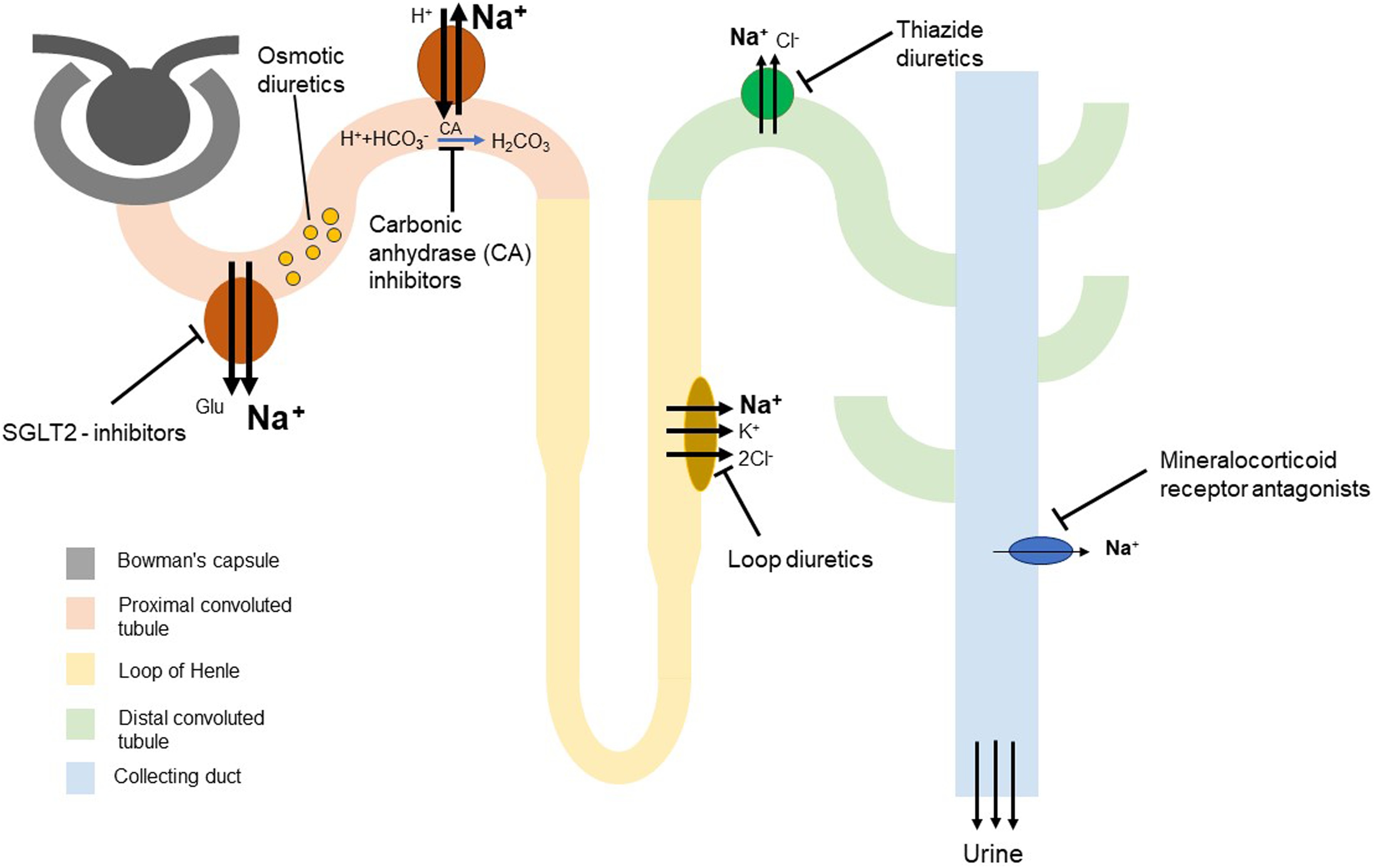
Diuretics - mechanisms of action. Approximately 60% of filtered sodium is reabsorbed in the proximal convoluted tubule (PCT) via various co-transporters. Sodium–glucose co-transporter 2 (SGLT2) inhibitors act at this site to reduce the reabsorption of both sodium and glucose. Carbonic anhydrase inhibitors (CAIs) indirectly decrease sodium uptake in the PCT by reducing hydrogen ion availability, thereby limiting sodium–hydrogen exchanger 3 (NHE3) activity. Loop diuretics inhibit the sodium–potassium–chloride cotransporter (NKCC2) in the thick ascending limb of the loop of Henle, where approximately 30% of sodium is reabsorbed. Thiazide diuretics act on the sodium–chloride cotransporter (NCC) in the distal convoluted tubule. In the collecting duct, mineralocorticoid receptor antagonists reduce the expression of epithelial sodium channels (ENaC). Under physiological conditions, both NCC and ENaC contribute only a minor portion to total sodium reabsorption. Osmotic diuretics increase tubular fluid osmolality, reducing water reabsorption, but have no effect on natriuresis.

The lymphatic system thus plays a central role in interstitial fluid clearance, but this process is relatively slow and depends on low-pressure gradients. When capillary leakage increases, the body can compensate by accelerating lymphatic flow, a mechanism known as interstitial washdown [15]. However, in critical illness, particularly in inflammatory conditions like sepsis or burn injury, high nitric oxide (NO) levels impair lymphatic smooth muscle contraction, leading to lymphatic failure [16,17]. In addition, numerous cytokines (e.g. interleukin 1β, tumor necrosis factor α, interleukin 6, etc.), and histamine reduce interstitial pressure to more negative values. Along with increased capillary permeability, this contributes to fluid accumulation in the interstitial space and consequently leads to hypovolemia, hypoalbuminemia, see Fig. 2 (Panel A) and oedema that often persists even after initial resuscitation. This is important to keep in mind when aiming to remove excess fluid. Because lymphatic clearance is slow in healthy conditions and even slower during critical illness, interstitial oedema can persist long after intravascular volume appears corrected. Therapeutic interventions such as diuretics or mechanical fluid removal (e.g., dialysis) may worsen hypovolemia and lead to hypotension, while fluid administration further aggravates oedema [18].

One example where lymphatic failure is particularly pronounced is in encapsulated organs like the kidney. In this setting, fluid accumulation leads to a rise in interstitial pressure, causing venous congestion, which promotes further capillary leakage and worsens interstitial oedema. The elevated interstitial pressure also compresses the intrarenal lymphatic vessels, blocking lymph outflow and further impairing interstitial fluid clearance [18]. This mechanism is commonly seen in acute kidney injury (AKI) and creates a particular problem, as the kidney is not only affected by oedema but is also the key organ responsible for eliminating excess fluid. Fluid overload in this context is strongly associated with increased mortality. Although biomarker-based screening strategies for AKI hold promise for earlier detection, they have not consistently improved clinical outcomes and are not yet integrated into routine care. In sepsis and other inflammatory states, aggressive fluid administration can exacerbate venous congestion and organ dysfunction, making it even harder for the body to mobilise and excrete the accumulated fluid [19].

## Fluid de-escalation

### Readiness for de-escalation

While the initiation of fluid therapy is well studied, evidence on fluid de-escalation is more recent and less specific. The ROSE concept (resuscitation, optimisation, stabilisation, evacuation) has been proposed as a structured framework to guide the phased management of shock, with fluid evacuation representing the final step [2]. At the same time, in daily ICU practice fluid de-escalation is usually guided by individualized assessment and real-time haemodynamic variables [20]. Nonetheless, a general framework for fluid de-escalation remains important to guide practice, particularly after initial resuscitation.

Understanding fluid de-escalation as the broader concept of reducing or withholding further resuscitative fluids, it should begin once the patient achieves haemodynamic stability. That is, in the absence of signs of tissue hypoperfusion such as elevated lactate, prolonged capillary refill time, or mottling. In this phase, fluids are no longer required to improve perfusion, and fluid balance targets can be set to neutral to prevent or reduce further fluid accumulation. Another approach to evaluating fluid status is the venous excess ultrasound score (VeXUS), a semi-quantitative, bedside ultrasound tool designed to assess the degree of systemic venous congestion. The VeXUS system categorizes congestion into four grades, with Grade 2 or higher typically indicating elevated right atrial pressure, increased mean systemic filling pressure, higher risk of acute kidney injury and adverse outcomes in critically ill patients. However, the evidence supporting its ability to predict outcomes in the general ICU population remains limited, and its accuracy can be affected by operator dependency and technical limitations [21]. Active fluid removal aimed at achieving a negative fluid balance is recommended in patients with clinical signs of fluid overload (pitting oedema, elevated central venous pressure, or a non-collapsing inferior vena cava) accompanied by organ dysfunction, particularly when spontaneous diuresis has failed [1,2]. A review of randomised trials in sepsis and acute respiratory distress syndrome (ARDS) showed that active de-resuscitation was usually guided by elevated filling pressures and the absence of vasopressor support [22]. In addition, extravascular lung water index (EVLWI) and pulmonary vascular permeability index (PVPI), measured via transpulmonary thermodilution, are validated quantitative markers of pulmonary edema and capillary permeability in ARDS [23]. Their potential role in informing fluid management strategies has been discussed, although robust evidence for outcome-guided de-escalation is limited.

Monnet et al. proposed a more pragmatic strategy aligned with clinical practice. They recommend initiating fluid removal via diuretics or ultrafiltration unless there is no clear fluid overload, or the patient remains preload responsive, an indicator that fluid removal is likely to be tolerated [3,24].

An alternative strategy involves earlier and more aggressive fluid offloading, even if it results in some degree of permissive haemodynamic instability, aiming to achieve an early neutral or negative fluid balance and potentially prevent fluid overload-related complications [25]. In the RADAR-2 trial, active de-resuscitation using diuretics began in patients with clinical signs of fluid overload and no contraindications, defined as norepinephrine or epinephrine doses exceeding 0.2 mcg/kg/min, use of multiple vasopressors, or lactate levels above 3.5 mmol/L [10]. The trial demonstrated that fluid removal was feasible and showed no significant differences in outcomes such as duration of mechanical ventilation, mortality, ICU length of stay, or new-onset AKI. However, limited sample size reduced the ability to interpret these findings [10]. It is worth mentioning that, in the subgroup analysis of patients with septic shock, mortality was significantly higher in the intervention group. However, the authors emphasised that these findings should be interpreted with caution owing to baseline imbalances between the two groups. In the Go Neutral trial, patients with circulatory failure on continuous renal replacement therapy (CRRT) were randomised to either ultrafiltration above 100 mL/h (adjusted by a hemodynamic-based protocol) or below 25 mL/h. The intervention group showed a greater negative fluid balance after 72 h [26]. However, certain aspects of the study warrant attention. Notably, patients in the control group had higher median ultrafiltration rates prior to randomisation, suggesting that the imposed target may have deviated from usual care and potentially limited further fluid removal. Furthermore, as with the RADAR-2 trial, the small sample size (*N* = 55) restricts the power to draw firm conclusions regarding clinical outcomes [26]. However, given that fluid overload may participate in multi-organ failure and is associated with worse outcome, aiming early negative fluid balance seems clinically sound and, based on current evidence, also safe [5]. Analogous to ventilator support, where extubation attempts are guided by an acceptable risk of failure to avoid prolonged ventilation, fluid management must also balance intervention risk (timely withdrawal) against the risks of complications due to persistent fluid accumulation [27]. In this context, the failure to actively reduce fluid volume despite clinical stability, may be deleterious [5,28]. Therefore, a more proactive and earlier transition to controlled de-escalation or de-resuscitation in some contexts should be considered to mitigate the harms associated with fluid overload.

### General principles

General principles of fluid de-escalation focus on minimizing unnecessary fluid administration. Analyses show that only a minority of fluids given are true resuscitation fluids. Depending on the study, maintenance and nutritional fluids account for up to 60% of total fluid intake, while drug-related fluids contribute up to 30% [29,30]. The first step in de-escalation is to prevent further fluid accumulation by restricting fluids to physiological needs and limiting fluids from other sources. In addition, all medications should be reviewed daily to assess whether conversion to oral or nasogastric administration is feasible [24].

### Diuretic therapy

In patients with residual urine output, diuretics are first-line agents for active fluid removal. Diuretic resistance may occur, but combination therapy targeting different nephron segments can enhance efficacy [31], see Fig. 2.

**Loop diuretics** are actively secreted into the proximal tubule and act on the thick ascending limb of Henle by inhibiting the Na^+^-K^+^-2Cl^−^ co-transporter (NKCC), where 25–30% of sodium is normally reabsorbed [32]. Thus, loop diuretics are very potent diuretics, and widely used in ICU settings [33]. Hypokalemia is a frequent adverse effect [31]. Continuous infusion may offer better tubular drug delivery and diuretic efficiency, though evidence on clinical outcomes such as mortality or hospital stay remains inconclusive [[34], [35], [36], [37]]. **Mineralocorticoid receptor antagonists** (MRAs) act in the distal nephron, sparing potassium while inducing modest diuresis due to limited sodium reabsorption at this site. They are often combined with other diuretics to counteract hypokalemia [31]. **Epithelial sodium channel** (ENaC) inhibitors, like MRAs, act distally, promote potassium retention, and induce mild diuresis [31]. **Thiazide diuretics** inhibit the Na^+^-Cl^−^ co-transporter in the distal convoluted tubule, causing moderate natriuresis. They are less effective in renal impairment and can cause hyponatremia [31,34]. Combined with loop diuretics, they may overcome resistance and enhance urinary output, though recent data show no significant sodium-lowering effect in ICU-acquired hypernatremia [34,38]. **Carbonic anhydrase inhibitors** (CAIs), notably acetazolamide, act in the proximal tubule to increase bicarbonate excretion, reduce sodium and water reabsorption, and induce mild diuresis and metabolic acidosis. They are useful in metabolic alkalosis and may potentiate loop diuretic response [31,34,39,40]. **Osmotic diuretics** (e.g., mannitol) increase filtrate osmolarity, reducing water reabsorption without directly affecting electrolyte transport. They are rarely used for diuresis due to risks of hyponatremia, hypokalemia, and fluid shifts causing pulmonary or cerebral oedema, especially with altered vascular permeability [31]. **Sodium-glucose co-transporter-2 (SGLT-2) inhibitors** reduce glucose and sodium reabsorption in the proximal tubule, causing mild diuresis [31]. While recommended in type 2 diabetes, heart failure, and chronic kidney disease, their role in active fluid removal on ICU is limited. In critical illness, they are generally well tolerated, but do not significantly affect major outcomes such as renal replacement therapy (RRT) initiation or ICU stay duration [41].

### Renal replacement therapy

Mechanical fluid removal is indicated in critically ill patients with anuric AKI, already established on RRT. In addition, it is recommended for those patients that are unresponsive to diuretics or at high risk from persistent or severe fluid overload [[42], [43], [44]].

RRT operates via convection and diffusion [45]. Convection involves solute and fluid transport across a semi-permeable membrane under a pressure gradient and predominates in hemofiltration, notably continuous veno-venous hemofiltration (CVVHF) [45]. Diffusion, governed by Fick’s law, enables solute clearance down a concentration gradient, as seen in hemodialysis (HD), peritoneal dialysis (PD), and continuous veno-venous hemodialysis (CVVHD) [45,46]. Since diffusion alone is inadequate for fluid removal, ultrafiltration via hydrostatic pressure is employed in both intermittent and continuous modalities [46,47].

**Intermittent hemodialysis (IHD)** facilitates rapid fluid removal with up to 5 L in hours, but may cause hemodynamic instability, limiting use in critically ill patients [48,49].

**Continuous renal replacement therapy (CRRT)**, including CVVHF, CVVHD, and continuous veno-venous hemodiafiltration (CVVHDF), is preferred in the ICU due to more gradual, better-tolerated fluid removal [50]. However, evidence comparing intermittent and continuous approaches remains inconclusive [51]. Some data suggest that more aggressive ultrafiltration in the setting of fluid overload may be associated with improved long-term survival, raising the possibility that effective fluid offloading could contribute to recovery [52]. Conversely, other studies have reported worse outcomes with higher ultrafiltration rates, indicating that excessive fluid removal may reflect or even exacerbate underlying physiological instability [53]. These contrasting findings point to the need for a nuanced approach to fluid management and emphasize the importance of prospective trials to disentangle causality from correlation.

**Slow low-efficiency dialysis (SLED)**, a hybrid of IHD and CRRT, allows gentler fluid removal, patient mobilization, reduced anticoagulation, and lower staffing requirements. It may be advantageous in selected ICU cases, though evidence of superiority is lacking [54].

**Peritoneal dialysis (PD)**, using the peritoneum as a natural membrane, regained interest during the COVID-19 pandemic due to limited RRT availability [55]. Cost-effective and infrastructure-sparing, PD is suitable for adults and children, particularly in resource-limited settings or when vascular access is problematic [42,56]. It is generally well-tolerated in unstable patients but has limited fluid removal capacity and is contraindicated after recent abdominal surgery or in intra-abdominal infection [42,56].

### Adjunctive therapies

**Hyperoncotic albumin** has been proposed as an adjunct to loop diuretics particularly when intravascular hypovolemia and hypotension coexist with interstitial oedema, and in patients with low serum albumin levels [57]. This approach aims to promote vascular refilling and restore effective circulating volume. However, its effect on volume restoration appears limited [58]. Moreover, high tubular albumin concentrations may reduce diuretic efficacy by lowering the free active drug fraction [34,59]. Overall, albumin is generally safe but costly, and current evidence regarding its benefit on net fluid balance remains limited and inconclusive [60]. **Compression bandages** may aid fluid removal by increasing vascular resistance and interstitial pressure, thereby reducing capillary leak and enhancing lymphatic drainage [[61], [62], [63]]. Observational data hints toward greater diuretic efficiency than hypertonic albumin [64]. Though simple to apply, they may cause skin injury or discomfort, especially if misused. Contraindications include peripheral arterial disease, right heart failure, neuropathy, and local infection. Frequent changes may reduce feasibility in resource-limited settings. **Hypertonic saline solution (HSS)** may enhance diuretic response by stimulating renal autacoids like TNF-α, affecting sodium handling and blood pressure regulation [65]. In acute heart failure, HSS with furosemide has been linked to increased diuresis, improved renal function, and better outcomes [66,67]. In post-cardiac surgery patients, a bolus of HSS increased urine output and reduced fluid balance [68]. Hypernatremia is the main adverse effect, particularly in combination with loop diuretics. While evidence supports its use in hyponatremic heart failure patients, its benefit in broader ICU populations remains unclear. **Vaptans**, such as tolvaptan, promote aquaresis by blocking vasopressin V2 receptors, increasing urine output without major electrolyte loss or renal impairment in critically ill patients [69]. In heart failure, tolvaptan enhances fluid and weight loss but offers limited symptomatic benefit, with hypernatremia as a potential risk [70].

## Special clinical contexts

Patients with **acute respiratory distress syndrome (ARDS)** are highly sensitive to fluid overload due to increased alveolar-capillary permeability and risk of non-cardiac pulmonary oedema. A conservative fluid strategy with early initiation of diuretics can improve oxygenation, shorten ventilation time, and reduce ICU stay [22,71,72]. When fluid resuscitation is unavoidable due to hemodynamic instability, early offloading with diuretics or RRT is essential once stability is achieved. Regular assessment of ELVI and PVPI can support decision in this patient population [23].

In patients requiring **high-volume fluid administration**, such as massive transfusion, parenteral nutrition, or high-dose infusions, early consideration of initiation of diuretics or mechanical fluid removal is warranted, later in patients with poor renal function [44]. Patients at risk for impending compartment syndrome after high-volume resuscitation may also benefit from timely offloading to prevent complications, such as tissue ischemia [8,9,42,].

In patients with **complex electrolyte and acid-base disorders,** fluid overload may worsen refractory electrolyte imbalances such as metabolic alkalosis, hyponatremia, or hypokalemia, especially during diuretic use. In these scenarios, mechanical fluid removal can be considered, to support both volume and electrolyte correction [8,42].

## Monitoring and defining endpoints of fluid de-escalation

As with the initiation of fluid de-escalation, there are no universally defined thresholds for when to stop. In general, fluid removal should continue as long as the patient is unable to offload fluid spontaneously and remains clinically fluid overloaded. Many critically ill patients exhibit persistent difficulty with fluid removal due to intravascular depletion, despite ongoing oedema, a result of complex fluid shifts commonly seen in this population [1]. As such, patients undergoing renal replacement therapy (RRT) may develop hemodynamic instability at any stage of treatment. It is characterized by an acute deterioration in cardiovascular function temporally associated with the initiation or continuation of RRT, most frequently manifesting as hypotension that necessitates therapeutic intervention. Thus, Fluid de-escalation should be viewed as a gradual weaning process, where slowing or temporarily pausing active de-resuscitation measures may allow interstitial fluid to mobilise into the intravascular space via lymphatic drainage [1,4]. To prevent adverse effects from overly aggressive fluid removal, repeated assessment of both macro- and microcirculatory function as well as ongoing evaluation of fluid responsiveness, are suggested [73]. While hypotension is a common phenomenon during dialysis and may arise from multiple mechanisms, including factors unrelated to intravascular volume depletion such as dialysate temperature, sodium balance, or extracorporeal removal of vasoactive agents, impaired tissue perfusion remains the clinically relevant concern, as it may contribute to new organ dysfunction even in the absence of profound arterial hypotension [73,74]. Although no gold standard exists for when to stop fluid de-resuscitation, proposed safety criteria include pulse pressure variation (PPV) or stroke volume variation (SVV) >15%, a positive passive leg raise (PLR) test, rising serum lactate >2.5 mmol/L, central venous oxygen saturation (ScvO₂) <65%, or mixed venous oxygen saturation (SvO₂) <70% [75]. Microcirculatory assessment complements these measures. Prolonged capillary refill time (CRT > 3 s) or low perfusion index (PI < 1.0) may indicate impaired peripheral perfusion, while near-infrared spectroscopy (NIRS) can provide additional information on tissue oxygenation and microcirculatory alterations, though its use is limited by technical complexity and variability between measurement sites (e.g., calf vs. vital organs such as brain or kidneys) [76]. When macro- or microcirculatory failure occurs, or once signs of fluid overload have resolved, the fluid balance target should shift toward neutrality [77]. This is particularly relevant for patients requiring prolonged RRT, in whom preventing recurrent fluid accumulation is essential [78,79]. In such cases, dialysis frequency may be reduced to alternate-day sessions [80].

## Future directions: toward phenotype-guided fluid de-escalation

Currently, fluid therapy in critically ill patients is guided by individualized assessments of fluid responsiveness, hemodynamic parameters (e.g., cardiac output), and filling pressures to determine the timing, dose, and cessation of fluid administration and active de-resuscitation [3]. However, emerging evidence suggests that distinct subgroups of patients may respond differently to fluid management strategies. In the Fluid and Catheter Treatment Trial (FACTT), which compared conservative versus liberal fluid strategies in patients with ARDS, no overall mortality difference was observed at 60 or 90 days [72]. Post hoc phenotyping of this cohort identified a hyperinflammatory subphenotype that derived significant mortality benefit from the conservative strategy (17% vs. 24%, *p* = .009), while the hypoinflammatory group experienced worse outcomes under the same strategy (50% vs. 40%, *p* = .004) [81]. Similar findings were confirmed in two additional analyses of the FACTT dataset [82,83]. These results highlight the potential of phenotype-guided fluid strategies to improve outcomes. However, evidence remains limited outside of ARDS.

Also in sepsis where endothelial breakdown plays a key role in the pathophysiology, there are currently no validated biomarkers available for routine clinical use to assess vascular leak or guide treatment. Future research should emphasize this gap, as it could lead to more targeted therapeutic strategies [84].

## Conclusions

Fluid management in critically ill patients goes beyond initial resuscitation and includes ongoing decisions about when and how to reduce fluid overload. Viewing fluid therapy as temporary organ support can help shift the focus toward timely reassessment and gradual withdrawal, much like other supportive therapies in critical care. Emerging evidence suggests that certain patient subgroups may respond better to specific strategies, supporting a more individualized, phenotype-based approach. Further research is needed to define safe and effective methods for fluid removal in the ICU.

CRediT authorship contribution statement

REB and ASM conducted the literature review and drafted the manuscript; AO designed the figures, and both CAP and AO critically revised the manuscript for important intellectual content.

## Funding

None.

## Ethical approval and consent to participate

N/A.

## Availability of data and material

N/A.

## Declaration of competing interest

REB, CAP, AO and ASM are affiliated with the Department of Intensive Care Medicine, which reports grants from Orion Pharma, Abbott Nutrition International, B. Braun Medical AG, CSEM AG, Edwards Lifesciences Services GmbH, Kenta Biotech Ltd, Maquet Critical Care AB, Omnicare Clinical Research AG, Nestle, Pierre Fabre Pharma AG, Pfizer, Bard Medica S.A., Abbott AG, Anandic Medical Systems, Pan Gas AG Healthcare, Bracco, Hamilton Medical AG, Fresenius Kabi, Getinge Group Maquet AG, Dräger AG, Teleflex Medical GmbH, Glaxo Smith Kline, Merck Sharp and Dohme AG, Eli Lilly and Company, Baxter, Astellas, Astra Zeneca, CSL Behring, Novartis, Covidien, Phagenesis, Cytel, and Nycomed outside the submitted work. The money was paid into departmental funds; no personal financial gain applied.
